# Conditioned Medium Protects Dopaminergic Neurons in Parkinsonian Rats 

**DOI:** 10.22074/cellj.2018.5343

**Published:** 2018-05-28

**Authors:** Malihe Nakhaeifard, Maryam Haji Ghasem Kashani, Iran Goudarzi, Arezou Rezaei

**Affiliations:** 1 School of Biology, Damghan University, Damghan, Iran; 2 Institute of Biological Sciences, Damghan University, Damghan, Iran

**Keywords:** Conditioned Medium, Dopaminergic Neurons, Parkinson’s Disease

## Abstract

**Objective:**

Adipose derived stem cells (ASCs) secrete numerous neurotrophic factors and cytokines in conditioned medium (CM), which protect neurons by its antioxidative and trophic effects. This research assesses the neuroprotective effect of ASC- CM on neurotrophins genes expressions and tyrosine hydroxylase positive (TH+) cell density in male Wistar rats lesioned by 6-hydroxydopamine (6-OHDA).

**Materials and Methods:**

In this experimental study, the groups consisted of lesioned and sham rats with unilateral injections of 20 µg of 6-OHDA neurotoxin and phosphate buffered saline (PBS) into the striatum, respectively. Another groups received intravenous injections of 3×10^6^ cells (ASCs group), 500 µl of CM (ASC-CM group) or medium [α-minimal essential medium (α-MEM) group)]. All rats underwent evaluations with the rotarod and apomorphine-induced rotation tests at 2, 4, 6, and 8 weeks post-injection. At 8 weeks we sacrificed some of the animals for real-time polymerase chain reaction (PCR) analysis, and evaluation of TH+cell counts.

**Results:**

We observed a significant decrease in contralateral turns to the lesions in the ASCs and ASC-CM groups compared to the neurotoxin lesioned or α-MEM groups at 8 weeks post transplantation. Cell and CM- injected rats showed a significant increase of staying on the rotarod compared to the lesion or α-MEM groups. Cell and CM-treated rats showed significant increases in the *NGF* and *NT3* genes, respectively, compared with the lesion group. Both treated groups showed significant increases in *BDNF* gene expression and TH^+^ cell density.

**Conclusion:**

The results suggested that ASCs and ASC-CM protected dopaminergic neurons through the expressions of neurotrophin genes.

## Introduction

Motor disorders of parkinson’s disease (PD) are caused by dopamine loss of corpus striatum as the result of nigrostriatal pathway degeneration (1, 2). Adult stem cells have been used to treat neurodegenerative diseases such as PD over the past few years. Transplanted cells have the capability to differentiate into neural cells or secret neurotrophic factors and create an appropriate microenvironment to protect residual dopaminergic neurons of the substantia nigra (SN) pars compacta. 

Adipose derived stem cells (ASCs) are a population of mesenchymal stem cells in the stromal or nonadipocyte compartment of adipose tissues. Intrastriatal transplantation of ASCs has been shown to protect against 6-hydroxydopamine (6-OHDA)-induced experimental PD in mice (3). Secreted neurotrophins, which modulate oxidative stress in the injured SN after cell therapy, are more effective than neural differentiation of transplanted cells to repair the nigrostriatal pathway (3, 4). The survival of transplanted cells increased when accompanied with nerve growth factor (NGF) injection. NGF played an antioxidative role to protect neurons (5). 

Human ASC transplantation stimulated angiogenesis and neurogenesis by secreting vascular endothelial growth factor (VEGF) and transforming growth factor- beta (TGF-ß) (6). According to low survival and tumorigenesis of transplanted cells, another therapeutic application of stem cell is the use of cultured ASCs conditioned medium (ASC-CM) to protect surviving neurons or stimulate renewal of axonal sprouting. The secretory factors of cultured stem cells are called the secretome, microvesicles, or exosome; the medium is CM (7). Numerous studies showed that stem cells secreted various growth factors into the CM, which had therapeutic effects on various diseases (6-14).

The neuroprotective effect of ASC-CM has been reported in an *in vitro* model of neuronal apoptosis (3). In addition, recent studies reported that secretory factors of stem cells might result in tissue repair and induce neurite outgrowth of PC12 cells *in vitro* (15). In this study, the degeneration of DAergic neurons of PD was the result of oxidative stress after 6-OHDA injection. CM could protect neurons from oxidative stress (16). Here, we intended to compare the effects of intravenous injection of ASCs and ASC-CM on motor impairment in a rat model; *BDNF, NGF* and *NT3* gene expressions; tyrosine hydroxylase positive (TH+) cell density at the injured sites. 

## Materials and Methods

In this experimental study, adult male Wistar rats that weighed 220-280 g were purchased from Pasteur Institute of Iran. They were kept in standard cages in a temperature- and climate-controlled room under a 12/12 hour light/dark cycle and had ad libitum access to water and food. The Research and Ethics Committee of Damghan University approved this experimental protocol. Animals were deeply anesthetized by an intramuscular injection of a mixture of ketamine hydrochloride and xylazine, and then placed in a stereotaxic frame. A total of 20 µg of 6-OHDA hydrobromide (Sigma-Aldrich, USA) in 4 µl of sterile saline that contained 0.2% ascorbic acid was injected into the right striatum by a 26-gauge Hamilton syringe (Hamilton, France) at a flow rate of 1 µl/minute. Stereotaxic coordinates from the bregma were: anteroposterior (AP)=-1.2 mm, mediolateral (ML)=-3.9 mm, and dorsoventral (DV)=-5 mm (17). The syringe was left in place for 5 minutes after the injection and then removed slowly to optimize toxin diffusion.

### Preparation and culture of rat adipose derived stem cells 

Fat tissues from the backs of the rats were cut under sterile conditions. The tissues were digested mechanically and enzymatically with 0.2% collagenase (Gibco, USA) (18). ASCs were extracted by adherence to the plastic flasks. We cultured the isolated cells with 10% fetal bovine serum (FBS, Gibco, USA) that contained a-minimal essential medium (α-MEM, Gibco, USA) and 1% penicillin/streptomycin (Gibco, USA). The cells were incubated at 37°C in air with 5% CO2. The culture medium was changed after the first 48 hours and every 3-4 days to remove any floating cells. When the culture reached 80% confluency (usually within a week), the cells were harvested by incubation with 0.25% trypsin and 0.02% EDTA (Merck) at 37°C for 3-4 minutes. Once harvested, the cells were sub-cultured (19). 

### Collection of adipose derived stem cell-conditioned medium

ASCs were cultured in α-MEM that contained 10% FBS. After four passages, 5×10^5^ plastic-adherent cells were washed three times with PBS, and cultured in serum-free medium for 72 hours to allow secretion of neurotrophic factors. ASC-CM was then collected, centrifuged at 2000 rpm for 5 minutes, filtered through a 0.22 mm syringe filter, and stored in a -80°C refrigerator (4, 16, 20).

### Treatment with adipose derived stem cells, ASC- conditioned medium and a-minimal essential medium 

At one week after the 6-OHDA lesion (18), the rats were anesthetized with a mixture of ketamine hydrochloride and xylazine. The ASCs (3×10^6^ cells, n=7) (21), ASC-CM (500 µl in four stages over a 2-month period, n=7) (22, 23), or α-MEM (500 µl in four stages over a 2-month period, n=7) were injected into the tail veins of the PD rats.

### Apomorphine-induced rotation test

We used the apomorphine-induced rotational test to determine the extent of the retrograde nigrostriatal lesion. The animals received intraperitoneal injection of 0.5 mg/kg apomorphine hydrochloride (Sigma- Aldrich, Germany) dissolved in 1% ascorbic acid, and 0.9% NaCl. The animals were placed on a cylinder (diameter: 28 cm) to monitor rotational asymmetry for 5 minutes. The net rotation asymmetry score was calculated by subtracting the total number of contralateral turns to the lesion from the total number of ipsilateral turns to the lesion prior to transplantation (1 week after the 6-OHDA injection) as well as at 2, 4, 6, and 8 weeks after transplantation (or equivalent times in the other groups). We chose only rats that exhibited at least 4 net rotations/minute (24, 25).

### Rotarod test 

Motor performance was evaluated on a Rotarod equipment (Hugo Basil, Biological Research Apparatus, Italy) with an accelerating protocol (26). The first 3 days of testing served as the training period. The animals underwent a 4 trial test under an accelerating protocol that went from 4 rpm to 40 rpm in 5 minutes, with a rest period for at least 20 minutes between trials. On the fourth day, using the same protocol, we recorded the latency to fall (24, 27).

### Immunohistochemical staining 

After 8 weeks, all animals underwent perfusion through the ascending aorta with 150 ml of 0.9% NaCl, followed by 500 ml of 4% paraformaldehyde in 100 mM phosphate buffer. The animals’ brains were extracted, post-fixed, and paraffinized. Next, they were cut at a thickness of 7 µm, starting at 12.3-13.7 mm and 7.9-9.3 mm from the anterior pole of the brain for the SN and striatum, respectively. A total number of six coronal sections per rat were obtained. Sections were deparaffinized and incubated in 0.1% Triton X-100 (Merck, Germany) for 10 minutes followed by 5% goat serum for 30 minutes at room temperature. 

The sections were then incubated with the primary antibody anti-TH (1:200, Millipore-AB152, USA) for 24 hours in a wet box at 4°C and then for 1 hour with goat anti-rabbit IgG-HRP (Santa Cruz Biotechnology, Germany) as the secondary antibody. The sections were washed twice with phosphate buffered saline (PBS) for 10 minutes after each step. When the staining reaction was completed, the tissue sections were sealed after washing and dehydration. The density of TH+ neurons of SN was measured with ImageJ software (28). All data were represented as mean ± SEM values with statistical significance set at P<0.05.

### Real-time polymerase chain reaction

After 8 weeks, all animals were killed and we removed their brains. The ipsilateral and contralateral striata (with respect to the lesion) were isolated for *BDNF, NT3,* and *NGF* mRNA evaluation. The noninjected side of each rat was used as the control. The samples were placed in RNX-plus (Cinnagen, Iran). Total RNA was isolated according to the manufacturer’s instructions. RNAquality was assessed by using a density ratio of 28S to 18S rRNA bands (29). A total of 1 µg total RNA was transcribed into cDNA according to the Thermo Scientific kit. Real- time polymerase chain reaction (PCR) was carried out with the Quantitect SYBR Green PCR kit (Jena Bioscience, Germany). Total reactions were done by using a Rotor Gene^TM^ 6000 (Corbett, India) Detection System. The no template control (NTC) was used as the negative control. The specificity of PCR products was confirmed by both melting curve analysis and agarose gel electrophoresis (19). The primers used in this study and *ß-actin* as the house-keeping (internal control) gene were listed (Table 1). The PCR conditions were as follows: initial activation at 95°C for 2 minutes, denaturation at 95°C for 15 seconds, annealing at 57°C for 30 seconds (*BDNF*), 62°C for 20 seconds (*ß-actin*), and 55°C for 30 seconds (*NT3* and *NGF*), extension at 72°C for 60 seconds, and amplification for 40 cycles. PCR reactions were run in duplicate using the reaction mixture that contained 1 µl cDNA, 0.5 µl forward primer (10 pM), 0.5 µl reverse primer (10 pM), 5 µl qPCR Green Master with low ROX (2x), and 3 µl RNAse-free water. Real time-PCR was performed in duplicate for each sample primer set. 

The mean of the three experiments was used as the relative quantification value. Relative gene expression was analyzed using the comparative Ct method, 2^-ΔΔCt^. All samples were normalized to the level of *ß-actin*, which was used as the internal control gene. A control cDNA was selected with the appropriate concentration. Successive dilutions of 4 different concentrations were used to draw a standard curve. PCR efficiency was determined for each gene according to the standard curves according to Rotor gene software. Amplification efficiencies (amplification curve) of all the genes were determined for each of the primers. Analyses were made per comparison of different samples’ C_t_ values (19). 

### Statistical analysis

We used SPSS software version 16, for data analysis (SPSS Inc., Chicago). Differences between groups were assessed by one-way ANOVA followed by the Tukey and LSD, least significant difference tests. P<0.05 was considered statistically significant. All values were expressed as mean ± SEM. 

## Results

### Passage-4 of adipose derived stem cells with spindle- shaped morphology 

Analysis of the cultured cells by inverted microscope showed fibroblast and spindle-like shaped passage-4 ASCs. In addition, we observed colonies of proliferative cells. 

### Intravenous administration of adipose derived stem cells and ASC-conditioned medium reduced rotational behavior of parkinson’s disease rats 

**Fig.1:**
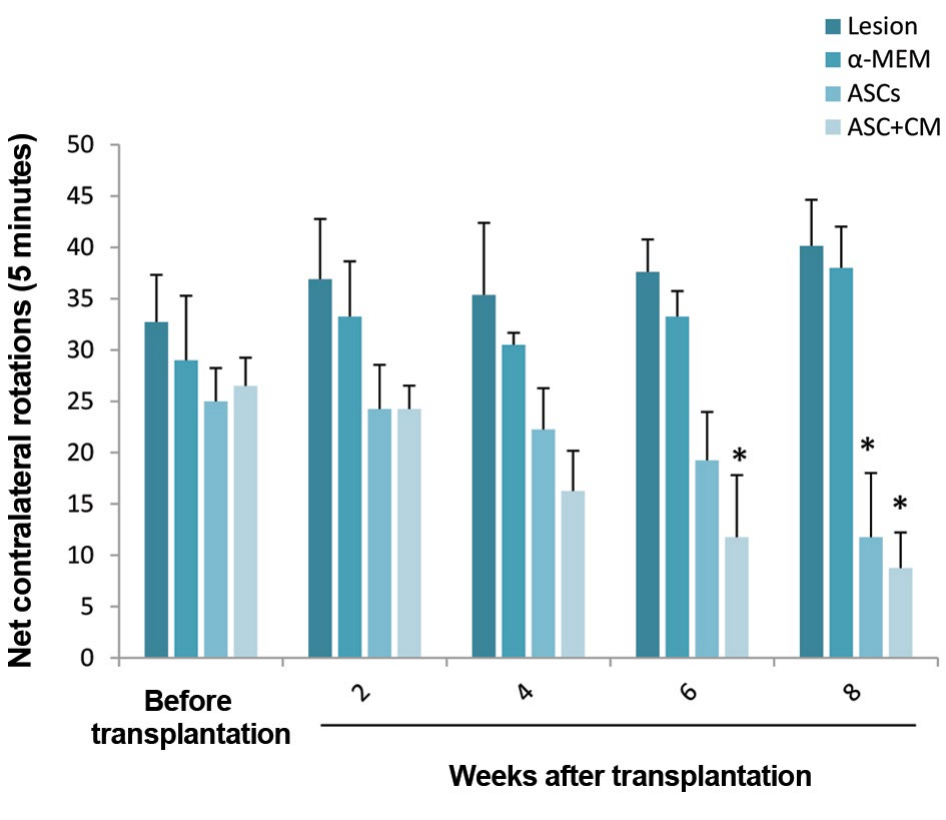
Number of apomorphine-induced rotation, before and after transplantation. *; P<0.05, asterisk denote significant difference from lesion and α-MEM groups. Data were expressed as mean ± SEM. ASCs; Adipose derived stem cells, CM; Conditioned medium, and α-MEM; a-minimal essential medium.

We did not detect any changes in the numbers of contralateral rotations between groups before, and 2 and 4 weeks after transplantation. At 6 weeks after transplantation, only the ASC-CM group showed a significant decrease in rotations compared to the α-MEM and lesion groups (P=0.01). In contrast, there was a significant lower number of net rotations in the ASC and ASC-CM groups compared to both the lesion (P=0.02) and α-MEM (P=0.01) groups at 8 weeks post-transplantation (Fig .1). 

**Table 1:** Primers used in the real-time polymerase chain reaction experiments


Gene	Primer sequence (5ˊ-3ˊ)	Primer size	Amplicon length (bp)	Reference

β-actin	F: GATTACTGCTCTGGCTCCTAG	21	147	(30)
	R: GACTCATCGTACTCCTGCTTG			
BDNF	F: GCCCAACGAAGAAAACCATA	20	405	(31)
	R: GATTGGGTAGTTCGGCATTG			
NT3	F: AGGTCAGAATTCCAGCCGAT	2017	181	(31)
	R: GTTTCCTCCGTGATGTT			
NGF	F: CCTCTTCGGACACTCTGGA	19	164	(31)
	R: CGTGGCTGTGGTCTTATCT			


### Intravenous administration of adipose derived stem cells and ASC-conditioned medium significantiy improved motor coordination on the rotarod test

**Fig.2:**
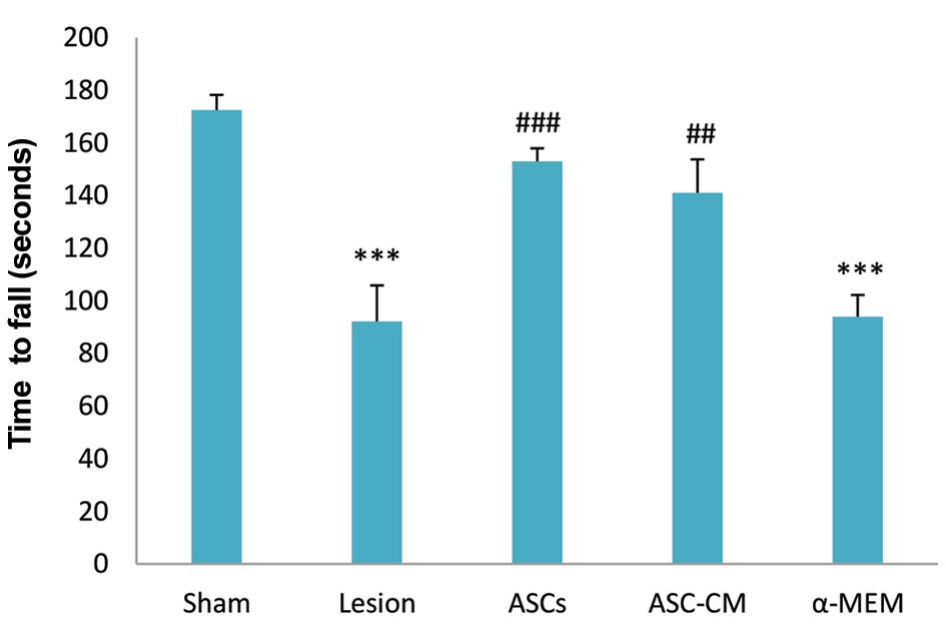
Effect of intravenous injection of ASCs and ASC-CM on motorbehavior at 8 weeks after transplantation. ###; P<0.000, ##; P<0.001 versus the lesion and α-MEM groups, ***; P<0.000 versus the sham group. Datawere expressed as mean ± SEM. ASCs; Adipose derived stem cells, CM; Conditioned medium, and α-MEM; a-minimal essential medium.

There was a significant decrease in time spent on the spinning rods of the rotarod in the lesion and α-MEM groups compared to the sham group (P=0.000). The ASCs and ASC-CM groups showed significant increases in time spent on the spinning rod compared to the lesion (P=0.001) and α-MEM (P=0.01) groups. The ASCs and ASC-CM groups showed no significant difference compared to the sham group at 8 weeks post-transplantation (Fig .2).

### Rats with adipose derived stem cells and ASC-conditioned medium transplantation showed better preservation of TH+ neuron density in the substantia nigra

**Fig.3:**
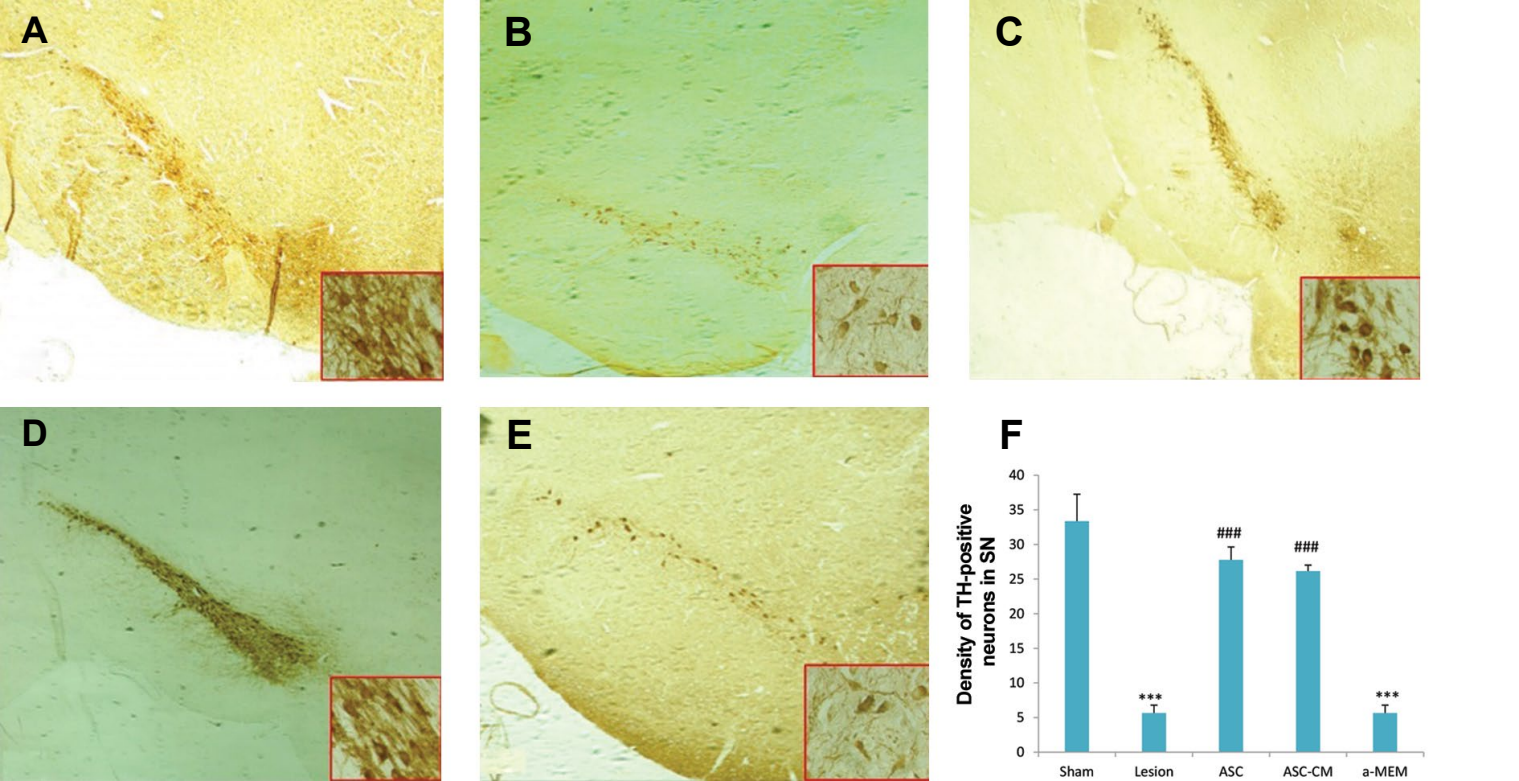
Immunohistochemical images of TH immunopositive neurons were shown. A. TH immunoreactivity in the SN of sham rats and B. Rats unilaterally lesioned with 6-OHDA alone, C. Rats treated with CM, or D. ASCs, or E. α-MEM (scale bar=100 µm). Small boxes in the corner of images indicates magnification of the SN region that shows dopaminergic neurons and their neuritis (×40), and F. The density of TH-positive neurons in SN of all groups. ***; P<0.000 versus the sham group and ###; P<0.000 versus the Lesion and α-MEM groups. Data were expressed as mean ± SEM. TH; Tyrosine hydroxylase, SN; Substantia nigra, 6-OHDA; 6-hydroxydopamine, ASCs; Adipose derived stem cells, CM; Conditioned medium, and α-MEM; a-minimal essential medium.

Immunohistochemical images of TH immunopositive neurons were shown (Fig .3A-E). There was a significant decrease in TH+ neuron density in the SN of the lesion and α-MEM groups compared to the sham group. We observed no significant difference between the treated and sham groups. The density of TH+ neurons in the ASCs and ASC-CM groups was significantly higher than the lesion and α-MEM groups (Fig .3F). 

### Neurotrophin gene expressions of the striatum

**Fig.4:**
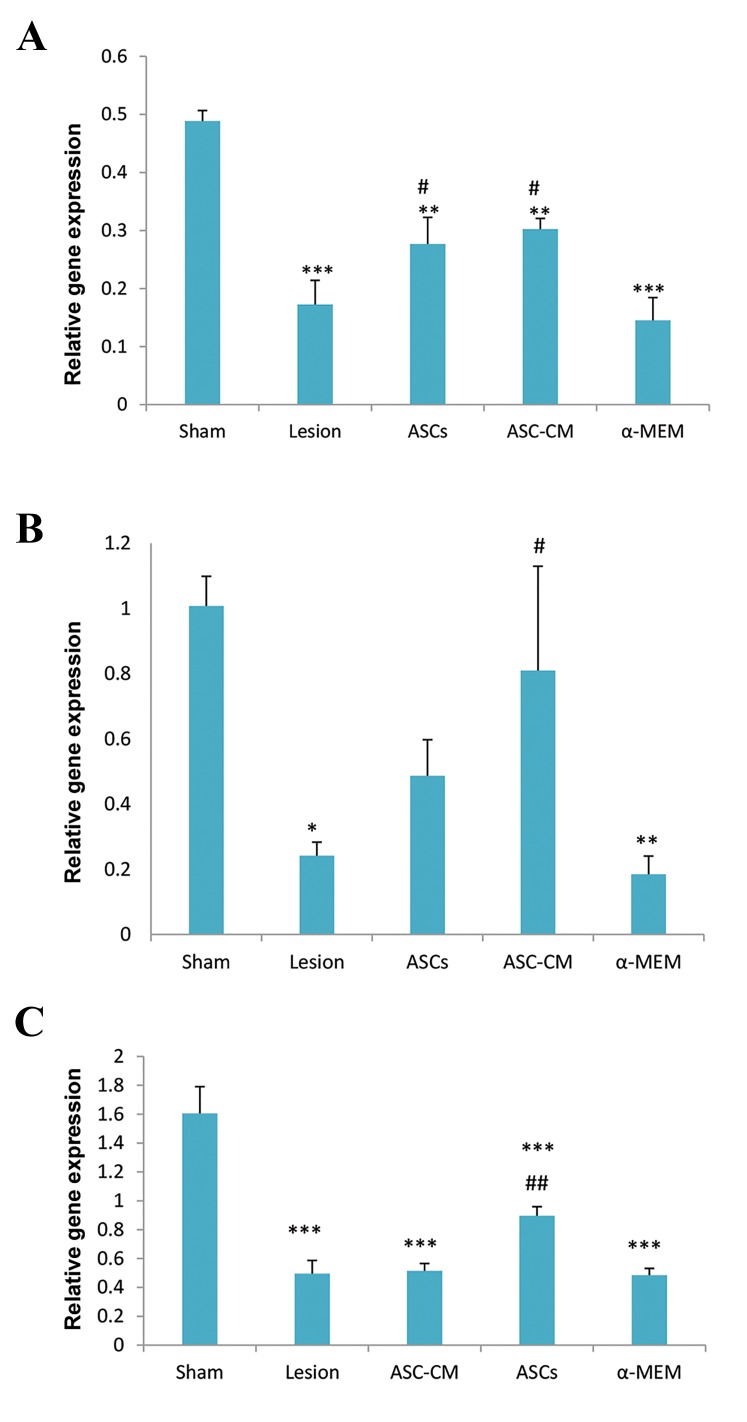
Effects of ASCs and ASC-CM injection on neurotrophin genes expressionof the striatum of parkinsonian rats. all groups showed a significant decreaseof BDNF gene expression in the striatum as compared to the sham group. ASCsand ASC-CM groups showed a significant increase of BDNF gene expression as compared to lesion and α-MEM groups (**; P<0.01, ***; P<0.001) versus the sham group. A. #; P<0.05 versus the lesion and α-MEM groups. NT3 geneexpression in lesion and α-MEM groups significantly decreased as comparedto sham group, and in ASC-CM group significantly increased as compared tolesion and α-MEM groups, B. *; P<0.05, **; P<0.001 versus the sham group, #; P<0.05 versus the lesion and α-MEM groups, and C. NGF gene expressionin all groups significantly decreased as compared to sham group, and NGF gene expression in ASCs group significantly increased as compared to lesionand α-MEM groups, ***; P<0.001 versus the sham group, ##; P<0.01 versusthe lesion and α-MEM groups. Data were expressed as mean ± SEM. ASCs; Adipose derived stem cells and CM; Conditioned medium, and α-MEM; a-minimal essential medium.

All groups showed a significant decrease in BDNF geneexpression in the striatum compared to the sham group. ASCs and ASC-CM groups showed a significant increase in geneexpression compared to the lesion (P=0.05) and α-MEM(P=0.02) groups. There was no significant difference betweenthe ASCs and ASC-CM groups. There was a significantincrease in expressions of the NGF and NT3 genes in the ASCs and ASC-CM groups compared to the lesion group (Fig .4).

## Discussion

In this study, we observed that intravenous administration of ASCs and ASC-CM of benefit and reduced apomorphine-induced rotations, as well as preserved TH-immunoreactive neurons. McCoy et al. (18) reported that the neuroprotective property of ASCs following transplantation was not related to its in vivo differentiation into neurons; instead, infused cells caused high amounts of neurotrophic factors (BDNF, GDNF, and NGF) mRNAs at the lesioned site. These factors have trophic and neuroprotective effects on nigral dopaminergic neurons (30, 31). Gu et al. (16) demonstrated that mesencephalic and cerebellar granule neurons could be protected against 6-OHDA-induced neurotoxicity by ASC-CM. This effect might be related to the neurotrophic factors of CM secreted by ASCs. The use of CM has several advantages compared to stem cells. CM can be manufactured, freeze-dried, packaged, and transported more easily. CM contains no cells; therefore, there is no need to match the donor and the recipient to avoid rejection problems. CM contains various growth factors and tissue regenerative agents, which are secreted by stem cells. However, intravenous injection of cells results in poor cell viability when passing through a thin syringe into the tail vein.

In the mature nervous system, neurotrophic factors play a major role in neuronal protection and the maintenance of cellular homeostasis; therefore, any change in their expression can be associated with neurodegeneration (32). Neurotrophic factors have been shown to activate receptor tyrosine kinases. Within neural precursors and neurons, the pathways regulated by tyrosine kinases include proliferation and survival, axonal and dendritic growth and remodeling, assembly of the cytoskeleton, membrane trafficking and fusion, and synapse formation and function. Recently, many studies on the neurotrophic factors have shown that they regulate each of these functions (33).

BDNF is a neurotrophic factor for dopaminergic neurons of the SN, the region affected by PD (30). Reduced expression of BDNF within the SN has been shown to cause the loss of dopaminergic neurons in PD. Indeed, postmortem studies of PD patients showed that a reduction in BDNF accompanied PD and BDNF was required to preserve neurons of the SN pars compacta (34). In this study, we assessed *BDNF* gene expression by real- time PCR. There was a significant decrese in *BDNF* gene expression in the striatal region of all groups compared to the sham group. However, ASCs and ASC-CM treated rats showed significant incereases in the mentioned gene expression compared to the lesion and α-MEM groups.

The expressions of *NGF* and *NT3* genes increased significantly in the ASCs and ASC-CM groups compared with the lesion group. It was suggested that transplanted cells that crossed the blood brain barrier (BBB) migrated into the lesioned zone and induced *NGF* gene expression. However, CM that contained NGF did not pass through the BBB. Although all treated groups showed behavioral improvement, maybe the cell or CM injection repaired the injured site by another mechanism such as induction of angiogenesis or neural differentiation. 

Possibly transplanted ASCs need adequate time to migrate from the peripheral vasculature into the damaged area to protect and restore destroyed dopaminergic neurons. Salinas reported that in PD, NGF like an antioxidant reduced ROS induced cell death due to 6-OHDA (35). It has been revealed that high sensitivity of dopaminergic cells to toxins or free radicals related to glutathione reduction, which was known as an intracellular antioxidant (36, 37).

As a result, we observed motor improvement. This treatment slows neurodegeneration progression. These reports have suggested that soluble factors of CM activate endogenous restorative and preserve the level of *BDNF* and *NT3* genes expressions and TH+ cells after a PD injury. The CM used in this experiment consisted of a serum-free media of the cultured cells for 72 hours. Therefore, it consisted of only the factors secreted by the cells. This strongly implied that the mechanism which underlies the observed protection was the presence of secreted neurotrophic factors. Hence, by changing the transplantation procedure, such as cell therapy accompanied by a CM injection, will reduce cell death and increase survival of the grafted cells. However, A more effective method should be designed to improve viability and provide an injected scaffold that protects cells from the damaging injection process. 

## Conclusion

The present data provided evidence that neuroprotection by ASC-CM was associated with stimulation of *BDNF* and *NT3* genes expression and TH^+^ neurons preservation. *BDNF* might be at least partly involved in neuroprotective effects. The significance of this study was that we first demonstrated which ASC-CM equally with ASCs could exert neuroprotection for 6-OHDA-exposed dopaminergic neurons *in vivo*. Secretome that contained CM has several advantages compared to stem cells and intravenous administration which would decrease damage to the patient. Clinical application of intravenous administration of ASC-CM for PD patients might be considered, although new methods are necessary. 
